# Barriers and facilitators to self-management in people living with a lower-grade glioma

**DOI:** 10.1007/s11764-024-01572-9

**Published:** 2024-03-21

**Authors:** Ben Rimmer, Michelle Balla, Lizzie Dutton, Sophie Williams, Vera Araújo-Soares, Pamela Gallagher, Tracy Finch, Joanne Lewis, Richéal Burns, Fiona Menger, Linda Sharp, on behalf of the Ways Ahead study team

**Affiliations:** 1 Population Health Sciences Institute, Newcastle University, Newcastle University Centre for Cancer, Ridley Building 1, Newcastle Upon Tyne, NE1 7RU England; 2 Faculty of Medical Sciences, Newcastle University, Newcastle Upon Tyne, England; 3 Newcastle Upon Tyne Hospitals NHS Foundation Trust, Newcastle Upon Tyne, England; 4 Centre for Preventive Medicine and Digital Health, Department for Prevention, Medical Faculty Mannheim, Heidelberg University, Heidelberg, Germany; 5 School of Psychology, Dublin City University, Dublin, Ireland; 6 Department of Nursing, Midwifery and Health, Northumbria University, Newcastle Upon Tyne, England; 7 Faculty of Science, Atlantic Technological University, Sligo, Ireland; 8 Health and Biomedical Strategic Research Centre, Atlantic Technological University, Sligo, Ireland; 9 School of Education, Communication and Language Sciences, Newcastle University, Newcastle Upon Tyne, England

**Keywords:** Barriers, Facilitators, Self-management, Lower-grade glioma

## Abstract

**Purpose:**

Self-management can have clinical and quality-of-life benefits. However, people with lower-grade gliomas (LGG) may face chronic tumour- and/or treatment-related symptoms and impairments (e.g. cognitive deficits, seizures), which could influence their ability to self-manage. Our study aimed to identify and understand the barriers and facilitators to self-management in people with LGG.

**Methods:**

We conducted semi-structured interviews with 28 people with LGG across the United Kingdom, who had completed primary treatment. Sixteen participants were male, mean age was 50.4 years, and mean time since diagnosis was 8.7 years. Interviews were audio-recorded and transcribed. Following inductive open coding, we deductively mapped codes to Schulman-Green et al.’s framework of factors influencing self-management, developed in chronic illness.

**Results:**

Data suggested extensive support for all five framework categories (‘Personal/lifestyle characteristics’, ‘Health status’, ‘Resources’, ‘Environmental characteristics’, ‘Healthcare system’), encompassing all 18 factors influencing self-management. How people with LGG experience many of these factors appears somewhat distinct from other cancers; participants described multiple, often co-occurring, challenges, primarily with knowledge and acceptance of their incurable condition, the impact of seizures and cognitive deficits, transport difficulties, and access to (in)formal support. Several factors were on a continuum, for example, sufficient knowledge was a facilitator, whereas lack thereof, was a barrier to self-management.

**Conclusions:**

People with LGG described distinctive experiences with wide-ranging factors influencing their ability to self-manage.

**Implications for cancer survivors:**

These findings will improve awareness of the potential challenges faced by people with LGG around self-management and inform development of self-management interventions for this population.

**Supplementary Information:**

The online version contains supplementary material available at 10.1007/s11764-024-01572-9.

## Introduction

In living with and beyond a cancer diagnosis, many people can face challenges with healthcare interactions, managing emotional distress, adjusting to a new normal, and re-establishing routine and social roles; engagement in self-management may help people meet and overcome these challenges [1]. Self-management in cancer is defined as “*awareness and active participation by the person in their recovery, recuperation, and rehabilitation to minimise the consequences of treatment, promote survival, health and well-being*” [2]. A growing evidence-base in people living with and beyond cancer suggests that quality-of-life, clinical (e.g. physical fitness), and health economic (e.g. reduction in healthcare utilisation) outcomes may be improved through self-management [3, 4]. It is, therefore, important to understand what factors influence whether someone can or does engage in self-management.

In people living with a chronic (non-cancer) illness, Schulman-Green et al. [5] identified five categories of wide-ranging factors that may present a barrier or facilitator to effective engagement in self-management, namely: ‘Personal/lifestyle characteristics’ (e.g. ‘*Motivation*’), ‘Health status’ (e.g. ‘*Symptoms/side-effects*’), ‘Resources’ (e.g. ‘*Financial*’), ‘Environmental characteristics’ (e.g. ‘*Community*’), and ‘Healthcare system’ (e.g. ‘*Relationship with providers*’). These factors are extensively supported by literature across chronic illnesses [6–10], neurological populations (e.g. multiple sclerosis (MS)) [11, 12], and some forms of cancer (e.g. breast, head and neck) [13–17].

Lower-grade gliomas (LGG) (e.g. grade 2 astrocytoma and oligodendroglioma) account for approximately 15% of gliomas, one of the most common types of brain tumour [18]. Unlike most common cancers (which tend to affect older adults), these tumours are typically diagnosed in adults in their 30 s and 40 s [19], are largely incurable, and often progress to a high-grade glioma [20], with a limited life expectancy of 5–15 years, depending on the subtype [19, 21]. People with LGG can experience substantial impacts on their daily lives (e.g. work, relationships, transport) [22], as a consequence of diverse, often co-occurring, symptoms and impairments. These can be both general cancer-related (e.g. fatigue, pain) and more tumour- and/or treatment-related (e.g. cognitive deficits, seizures) and can persist long-term [23].

The specific challenges faced by people with LGG may nuance the factors evidenced to influence self-management. For example, the psychological burden of living with an incurable condition [24] may influence one’s motivation to self-manage. Only one study appears to have explored barriers to self-management in people with brain tumours, finding that knowledge of their condition and available support were barriers to support service utilisation [25]. However, this study included all types of primary brain tumours with varying prognoses and focused specifically on access to support services. We have previously reported that people with LGG engage with a wide range of self-management strategies, such as self-monitoring and acquiring information [26]. To understand how best to encourage and support people with LGG to self-manage, it is important to have a comprehensive understanding of the factors that influence their engagement with self-management across a range of contexts in day-to-day life [27].

This analysis, therefore, aimed to identify and understand the barriers and facilitators to self-management in people with LGG, with the intention of helping to inform the development of self-management interventions for this population.

## Method

### Design

This qualitative study, part of the multi-method Ways Ahead project [28], used semi-structured interviews to generate data on experiences of self-management in people with LGG across a range of contexts in day-to-day life. The analysis reported here highlights the factors that may influence the self-management strategies used by people with LGG that we have reported elsewhere from this dataset [26]; the two papers are thus complementary. Ways Ahead was reviewed and approved by the Wales Research Ethics Committee (REC ref: 20/WA/0118).

### Participants and recruitment

Potentially eligible people with LGG were identified through collaborating National Health Service (NHS) sites and the Brain Tumour Charity’s networks. Participants were aged ≥ 18 years when diagnosed with a grade 2 astrocytoma or a grade 2 or 3 oligodendroglioma [29]. Individuals resided in the United Kingdom (UK), and were stable under observation, or had completed primary treatment. Non-English speakers or those with severe psychosocial problems – determined by healthcare professionals at collaborating NHS sites – who were at risk of further distress by participating, were excluded. Purposive sampling was used to ensure a range of ages, both sexes, diagnoses, and times since diagnosis.

Healthcare professionals at collaborating NHS sites identified people with LGG from their medical records and provided them with an information sheet by post or during a clinic visit. A researcher (BR) advertised the study on the Brain Tumour Charity’s networks with the information sheet attached. The information sheet included a brief introduction to the researchers conducting the interviews. People with LGG were asked to register their interest by contacting the study team; BR and LD subsequently called to confirm eligibility, afford the opportunity to ask questions, and, if willing, arrange a convenient interview date and time. A follow-up call could be requested if the individual needed more time to process the information. Recruitment was carried out between August 2020 and May 2022.

### Data collection

Trained and experienced in qualitative research, BR (male, MSc) and LD (female, PhD) conducted all interviews remotely, using video-conferencing software (e.g. Zoom or Microsoft Teams) or telephone. Cognitive or communication impairments can influence people with LGGs’ ability to retain, process, and respond to questions. To support the participation of people who may have had these impairments, we provided a topic overview in advance, and allowed ample time to consider and respond to each question during interviews.

Immediately prior to each interview, audio-recorded consent was acquired. Basic demographics (e.g. sex, age, employment and relationship status) and clinical and tumour-related information (e.g. diagnosis, tumour location, and treatment) were also collected. Participants recruited through the Brain Tumour Charity were asked for their main treating hospital and consultant. The treating hospital of every participant was contacted to confirm clinical and tumour-related information; where confirmation could not be obtained, this information was self-reported.

Interviews were semi-structured following a topic guide (*Online resource 1*), which comprised open questions informed by the literature. Appropriate modifications were made following discussions with a patient and public involvement panel of people affected by brain tumours, and the study team healthcare professionals (JL, SW). Throughout data collection, the topic order varied, influenced by what the participant chose to speak about.

Interviews commenced with a broad reflection on life following the LGG diagnosis. Participants’ experiences of how they were impacted by the tumour and its treatment (e.g. cognitive, physical, psychological) and how this affected daily life (e.g. work, relationships, and finances) were then explored. We asked probing questions across each area for what people did to self-manage living with the tumour, and what helped and hindered them to do this. Throughout the interview, participants could raise any additional issues they perceived as important; new issues raised were explored in subsequent interviews. Following each interview, a post-interview sheet detailing available charities and helplines was provided, alongside offer of a £20 voucher to thank them for their time. Interviews were audio-recorded, averaging 102 min in length (range 54 to 167 min).

### Data analysis

Each interview was transcribed verbatim and anonymised. We applied the framework method [30] to identify and understand the barriers and facilitators to self-management in people with LGG.

Analysis commenced with inductive open coding in accordance with the initial steps of an inductive thematic analysis [31]. Following independent familiarisation, BR and MB, both trained in qualitative analysis, generated initial codes for a sample of transcripts (n = 6 of 28). BR and MB discussed similarities and resolved differences to refine the coding frame. BR coded the remaining transcripts and discussed codes and uncertainties with MB and LS, as coding progressed. This stage of analysis occurred in parallel with data collection. Recruitment ceased when reasonable data sufficiency was reached; this was determined by the judgement of the research team that sufficient data had been generated to support and understand the barriers and facilitators to self-management in people with LGG [32].

These codes were then deductively mapped to Schulman-Green et al.’s pre-existing framework of 18 factors across five categories influencing self-management [5], namely: ‘Personal/lifestyle characteristics’ (e.g. ‘*Motivation*’), ‘Health status’ (e.g. ‘*Symptoms/side-effects*’), ‘Resources’ (e.g. ‘*Financial*’), ‘Environmental characteristics’ (e.g. ‘*Work*’), and ‘Healthcare system’ (e.g. ‘*Access*’). This charting was conducted by BR and, to enhance trustworthiness, checked by MB; any disagreements were discussed to reach consensus. During this stage, we remained alert to any new factors influencing engagement with self-management that were not included within the existing framework, though all the data fit, so no new factors were added. Below, we report how our participants’ experiences related to Schulman-Green et al.’s framework [5], with illustrative quotes throughout.

## Results

### Participant characteristics

Thirty-five of 39 people with LGG that registered their interest were eligible; exclusion reasons included: non-completion of primary treatment (n = 2), ineligible diagnosis (n = 1), resided outside the UK (n = 1). We purposively selected 28 people with LGG for interview (recruitment routes: The Brain Tumour Charity, n = 18; NHS sites, n = 10). Sixteen participants were male (Table 1). At interview, mean age was 50.4 years (median 52 years, range 22–69 years) and mean time since diagnosis was 8.7 years (range 1–18 years). Diagnoses were: grade 2 astrocytoma (n = 9: IDH1-mutant, yes n = 6, no n = 1, unknown n = 2; 1p/19q codeletion, no n = 7, unknown n = 2), grade 2 oligodendroglioma (n = 10: IDH1-mutant, yes n = 7, no n = 2, unknown n = 1; 1p/19q codeletion, yes n = 9, unknown n = 1), and grade 3 oligodendroglioma (n = 9: IDH1-mutant, yes n = 6, no n = 1, unknown n = 2; 1p/19q codeletion, yes n = 7, unknown n = 2).

**Table 1:** Lower-grade glioma participants’ characteristics at time of interview

Characteristic	n	Characteristic	Mean (range)
***Diagnosis***^***a***^		***Time since diagnosis (years)***^***a***^	8.7 (1–18)
Grade 2 oligodendroglioma	10	***Time since radiotherapy (years)***^***a,c***^	6.9 (0.7–17.8)
Grade 3 oligodendroglioma	9	***Time since chemotherapy (years)***^***a,c***^	3.4 (0.1–13.5)
Grade 2 astrocytoma	9	***Full-time education (years)***	15.8 (11–20)
***IDH-mutation status***^***a***^		***Sex***	**n**
Yes	19	Female	12
No	4	Male	16
Unknown	5	***Age***	
***1p/19q codeletion status***^***a,b***^		≤ 40	4
Yes	16	41–50	8
No	7	51–60	11
Unknown	5	> 60	5
***Treatment***^***a***^		***Dependents***	
Surgery	28	None	18
Radiotherapy	22	One	3
Chemotherapy	17	Two	6
***Tumour location***^***a***^		Three	1
Frontal	18	***Employment status***	
Temporal	3	Full-time employee	8
Parietal	3	Part-time employee	4
Overlapping regions	3	Retired	4
Unknown	1	Medically retired	6
***Tumour laterality***^***a***^		Unable to work	6
Right hemisphere	13	***Relationship status***	
Left hemisphere	15	Married	21
Dominant hemisphere	13	In a relationship	3
Non-dominant hemisphere	15	Single	2
		Widowed	2

^a^Clinical and tumour-related details were self-reported for eight participants

^b^All participants with 1p/19q codeletion were people with oligodendroglioma; all participants without 1p/19q codeletion were people with astrocytoma

^c^Time since radiotherapy and chemotherapy was not available for two participants

### Factors influencing self-management

**Fig. 1:**
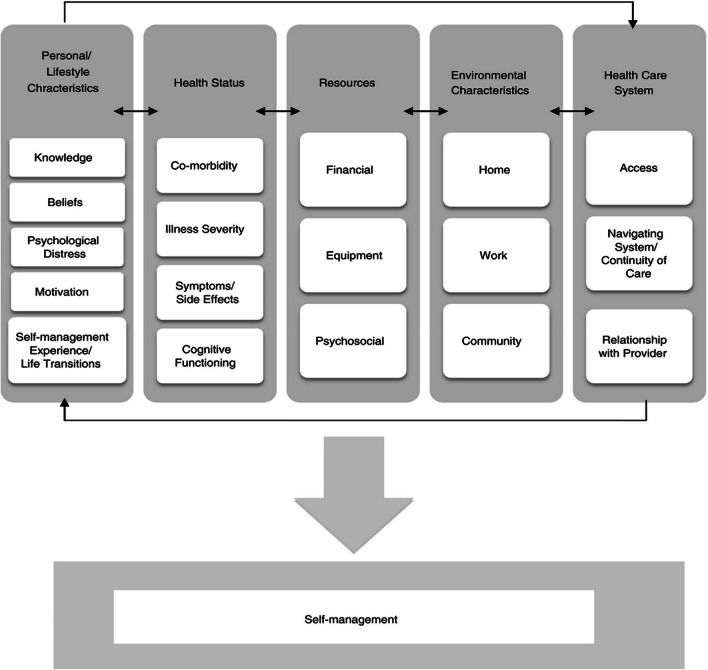
Factors influencing self-management. ^a^Re-used with permission from the Copyright Clearance Center: Wiley, Journal of Advanced Nursing, [5]. A metasynthesis of factors affecting self-management of chronic illness

Our data suggested extensive support for all five categories and all 18 factors influencing self-management in Schulman-Green et al.’s framework [5] (Fig. 1); additional supporting quotes can be found in Table 2. Individual factors that were spoken about most extensively across most participants were: ‘*Symptoms/side-effects*’ (within ‘Health status’), ‘*Psychosocial*’ (within ‘Resources’), ‘*Community*’ (within ‘Environmental characteristics’), ‘*Navigating system/continuity of care*’, and ‘*Relationship with provider*’ (both within ‘Healthcare system’).

**Table 2:** Additional supporting quotes for all categories and factors influencing self-management, with participant ID, age group at interview, sex, and tumour type

Categories and factors	Illustrative quotes
**Personal/lifestyle characteristics**	
*Knowledge*	• “It’s an oligodendroglioma and that that can potentially be dealt with again, you know, if it got more severe, that there are other treatments that it should respond to. So, yeah. So, I think that is the good thing about having that extra information” – Pa10 (aged < 40, female, grade 2 oligodendroglioma) • “I thought I was having a seizure**,** so we were panicking because I hadn’t had a seizure since before my operation. Then it transpired that it was actually an aura before a migraine and I had no idea, clue that you could have auras before migraines because I’d always had migraines without auras.” – Pa36 (aged 41–50, female, grade 2 astrocytoma)
*Beliefs*	• “I’m still not convinced I’ve necessarily fully accepted my diagnosis because some days I’m just, “I don’t have a brain tumour. I’m sure I don’t have a brain tumour.” I know I do but sometimes it’s hard just to be forced into living a different life as such.” – Pa36 (aged 41–50, female, grade 2 astrocytoma) • “There was a huge sense of not knowing what was going to happen and a loss of any kind of feeling of control over my life.” – Pa29 (aged 51–60, female, grade 3 oligodendroglioma) • “I’m not like the same as what I was before. I was struggling with that because I’ve got mobility problems and the right sided weakness. Obviously, I am working on that but it’s just slow progress. I feel that I’ve reached a plateau**,** but I don’t want to [pause] give up if you like.” – Pa22 (aged 41–50, female, grade 2 astrocytoma)
*Psychological distress*	• “Sometimes the fear of death and the fear of what’s going to happen next and brain surgery for a second time potentially, it gets on top of you and not being able to life your life as fully as you hoped you would.” – Pa36 (aged 41–50, female, grade 2 astrocytoma) • “You wouldn’t be totally comfortable planning anything beyond three months down the line or whenever the next scan is I guess and that obviously longer term, you just don’t know what it’s going to look like. I think anything beyond a three-month time period really.” – Pa40 (aged < 40, female, grade 2 astrocytoma) • “I just felt I was getting squeezed and squeezed and squeezed and I was going to break to a point but slowly but surely the layers are coming off.” – Pa37 (aged 51–60, male, grade 2 astrocytoma)
*Motivation*	• “Talking from a man’s perspective, going to a counsellor feels like defeat…it feels like you’ve accepted defeat**,** and it shouldn’t feel like that. Like, it’s taken me a year to get a counsellor. But that’s because I’ve only just now got the courage to talk about it, whereas I feel like if it was easier then I would talk about it sooner.” – Pa9 (aged < 40, male, grade 2 astrocytoma) • “Emotionally, I try and look at things just as positively as I can. I have two small kids. I’ve got my wife. I can’t just go on moping about stuff**,** so I just try and stay positive emotionally. I just try and stay positive.” – Pa33 (aged 41–50, male, grade 2 oligodendroglioma) • “I’ve always felt quite well supported by the health professionals and also brave enough that I can ring them and say I needed a bit of information on that.” – Pa29 (aged 51–60, female, grade 3 oligodendroglioma)
*Self-management experience/* *life transitions*	• “I didn’t have a job. I lost my home. I had to move house. My marriage broke down. So, loss was a huge thing.” – Pa17 (aged 51–60, female, grade 3 oligodendroglioma) • “One of the lessons that I need to learn and to remind myself of, is the importance of self-care which can be done in a way that is not selfish in orientation but needful to make the most of. You can’t give to the world if you’re not giving to yourself in a way” – Pa14 (aged > 60, male, grade 2 oligodendroglioma) • “One 10 k a week for a year. And that doesn’t sound like a lot because I used to run 10ks every day. And I thought that will be easy. But I didn’t take into account that I started it when I was just starting my chemotherapy. So, it was hard. I thought I was indestructible**,** but I realised I wasn’t.” – Pa11 (aged 51–60, male, grade 2 oligodendroglioma)
**Health status**	
*Co-morbidity*	• “There is apparently a school of thought that the thyroid medication I’m on [for thyroid cancer] can be bad for brain tumours.” – Pa40 (aged < 40, female, grade 2 astrocytoma) • “Yesterday, I could hardly walk**,** and some people say it could be your age, you could be arthritis and it could be this and it could be that. I don’t want to overthink it or be a burden to anybody or on the other hand I’m stabbing in the dark for an answer.” – Pa14 (aged > 60, male, grade 2 oligodendroglioma) • “I feel that my memory isn’t what it was. That might be tumour removal. It might be something else. It might be age. It might be self-abuse, who knows. But certainly**,** my memory isn’t what it was.” – Pa35 (aged 41–50, male, grade 2 astrocytoma)
*Illness severity*	• “To be honest, in terms of what it actually means we can and can’t do, it has very… it’s very hard for me to actually point to anything I can’t do. Driving is probably the one thing that I, you know… that is unusual that I can’t do.” – Pa3 (aged 41–50, male, grade 2 oligodendroglioma) • “I am lucky. I really am lucky, I've seen other people with brain tumours who are far worse than I am as far as the impact it had on their life and the life of their loved ones and the disability that they're having to cope with” – Pa15 (aged 51–60, male, grade 2 astrocytoma)
*Symptoms/side-effects*	• “It’s a bit worse for me now because I like to walk and I can’t walk now whereas before I’d walk as far, I’d walk on my frame and still got as far as I could before because then you get all the fresh air and the trees.” – Pa30 (aged > 60, male, grade 3 oligodendroglioma) • “Seizures. I’m not allowed to go swimming, and I used to be an avid swimmer. I used to love it. So, that’s something else that I can’t do now.” – Pa9 (aged < 40, male, grade 2 astrocytoma) • “I went through a few boxes just to check and I was absolutely done in. I went to bed at 2 o’clock in the afternoon, yesterday because I’d sorted through four boxes.” – Pa19 (aged 51–60, male, grade 3 oligodendroglioma)
*Cognitive functioning*	• “[medication] goes in the medicine pot and it sits on the kitchen bench so I can see it all the time because if I didn’t see it, I would probably forget to take it.” – Pa34 (aged > 60, female, grade 2 oligodendroglioma) • “This has gone on for so long that there’s not much I can remember how to cook, now. I mean, I probably would be able to cook a sandwich. You know, cook the sausages. I’d be able to put stuff in the grill, but I wouldn’t remember how to do, like, a proper meal.” – Pa25 (aged 41–50, male, grade 2 oligodendroglioma)
**Resources**	
*Financial*	• “I saw my naturopath… and again, I was paying, like, £60 a session. So, I saw him for a while, but I couldn’t… and then in between there was always things to buy and it just got too expensive.” – Pa17 (aged 51–60, female, grade 3 oligodendroglioma) • “I knew I needed to get the mortgage away from me to give me a chance to survive on half a wage. So we managed that with savings and things like that. So the house we live in now, we own. We don’t have a mortgage and that’s a big help.” – Pa30 (aged > 60, male, grade 3 oligodendroglioma)
*Equipment*	• “This shoulder bracelet I’ve got on has already… it makes me walk straighter, and it… and I think, if I can get my shoulder right, I’ll probably get my elbow right, and then I’ll probably get my wrist right. If I get my wrist right, I stand a chance of getting my fingers back.” – Pa13 (aged 51–60, male, grade 3 oligodendroglioma) • “I’ve got a little plastic box thing in the kitchen where I put out the medicines for about the next week or the next five days or something, morning and evening.” – Pa28 (aged > 60, male, grade 2 astrocytoma)
*Psychosocial*	• “I only go to the pub, for example, if I’m going with a friend, I could not go on my own to somewhere like that [because of the seizures].” – Pa25 (aged 41–50, male, grade 2 oligodendroglioma) • “I know she’d [my daughter] be supportive if I said I couldn’t do something. As she lives nearby she will be supportive as indeed with my son, that’s good.” – Pa5 (aged 51–60, male, grade 2 oligodendroglioma)
**Environmental characteristics**	
*Home*	• “We were in a larger house before, so we basically downsized a bit. And so, as we moved in, we thought… we worked out that walk-in showers were essential, so we got a walk-in shower.” – Pa13 (aged 51–60, male, grade 3 oligodendroglioma)
*Work*	• “They said they’ll never push me. They’ll never well yes, push me to, “When will you go full time?” No, they won’t do that. At some point they probably will but at this precarious time at the minute, no, they’ve been more than supportive.” – Pa18 (aged 51–60, female, grade 3 oligodendroglioma) • “At times it’s very, very, very stressful, to the point, with the condition I have and the drugs I take, my bosses have said, “Just go into the yard, have a walk round, then come back.” I’ll come back settled and I’ll just fly into it again.” – Pa37 (aged 51–60, male, grade 2 astrocytoma)
*Community*	• “I don’t see a lot, where I am. Because when I’m on this information group, there are a lot of people going to meet-ups and things, and I did tell The Brain Tumour Charity that there is nothing round here. The last time I looked, the closest one to me was on the other side of [place], and I can’t travel very well.” – Pa20 (aged 41–50, female, grade 3 oligodendroglioma) • “We live near major bus links really so it just wasn’t a problem. The only places I used to go were [City] to do some shopping or go for a drink or whatever, my parents’ house and the nursery and primary school. The nursery and primary school are right next to each other and they’re on a bus route where the bus is literally outside our house” – Pa33 (aged 41–50, male, grade 2 oligodendroglioma) • “There’s been times when I’ve been in amongst a crowd of people and have a seizure. I’ve had somebody say, “Get off the bus.” You know, I’ve had one where the driver said, “Get off the bus.”” – Pa25 (aged 41–50, male, grade 2 oligodendroglioma)
**Healthcare system**	
*Access*	• “In an ideal world you’d have all of this information at your fingertips because anybody with a brain tumour doesn’t want to receive a plethora of post with loads of paper because you’re still getting to grips with the fact that you have a debilitating, longstanding illness.” – Pa18 (aged 51–60, female, grade 3 oligodendroglioma) • “I’ve had to seek it out. There’s nothing upfront that says, “This is what you’ve been diagnosed with. This is what you can expect. This is what we can do for you.” … I’ve had to go and look for it.” – Pa20 (aged 41–50, female, grade 3 oligodendroglioma) • “I had to pay for it because nothing was available apart from the counselling through Macmillan and a few treatments like Reiki through Macmillan. everything else was paid for that I did.” – Pa17 (aged 51–60, female, grade 3 oligodendroglioma)
*Navigating system/* *Continuity of care*	• “I understand it’s the patient’s responsibility to negotiate. But they should at least be given the tools to allow them a fighting chance…I think they should point you in the right direction.” – Pa9 (aged < 40, male, grade 2 astrocytoma) • “What constitutes primary treatment? Brain tumour charity talk about adjuvant or primary. I’ve got a letter from [consultant] describing my radiotherapy and chemotherapy as adjuvant. So I thought, aha, two years from the surgery. So, I applied, I got back a large envelope from the DVLA saying: “No, no, it’s two years from the end of primary treatment.” – Pa16 (aged > 60, male, grade 3 oligodendroglioma)
*Relationship with provider*	• “I remember one of these was very, kind of, stand-offy – he didn’t even make eye contact with me. Another person, I kind of talked about having a lot of migraines and I was worried about the migraines, and was that something. And they said, “Well, you know, you’ve had brain surgery. You’re sure to have headaches, aren’t you?”…I thought, “That’s not a particularly helpful thing to say.” – Pa3 (aged 41–50, male, grade 2 oligodendroglioma) • “As soon as we went in there she was almost like, “I want to put your mind at rest about this,” kind of thing. Even though it’s become more serious… I think she even said, “The treatment for this we can get for you is better.” So**,** it’s worse but we can do more for you for it kind of thing. Whatever she said was really reassuring.” – Pa32 (aged 41–50, female, grade 3 oligodendroglioma)

Several factors influenced self-management on a continuum; whether a factor was a barrier or facilitator to self-management was determined by where the individual fell on the continuum. For example, sufficient financial resources was a facilitator, whereas a lack thereof, was a barrier to self-management. Within each category, this applied most prominently to the following individual factors: ‘*Knowledge*’ (within ‘Personal/lifestyle characteristics*’*), ‘*Symptoms/side-effects*’ (within ‘Health status’), ‘*Psychosocial*’ (within ‘Resources’), ‘*Community*’ (within ‘Environmental characteristics’), and ‘*Relationship with provider*’ (within ‘Healthcare system’).

### Personal/lifestyle characteristics

The five factors that participants spoke about within ‘Personal/lifestyle characteristics’ were ‘*Knowledge*’, ‘*Beliefs*’, ‘*Psychological distress*’, ‘*Motivation*’, and ‘*Self-management experience/life transitions*’.

Several participants highlighted the importance of knowledge for understanding how to self-manage their condition. For some, knowledge and awareness of potential treatment pathways provided reassurance that consequences of their condition could be managed. Alternatively, not knowing what symptoms and impairments they might experience meant that some participants either did not seek the necessary information and support or found themselves distressed when a symptom (e.g. seizure) spontaneously occurred.

“If you don’t know what to ask for, you don’t know… if you don’t know that people can get fatigued, you’re not going to ask about fatigue.” – Pa33 (aged 41–50, male, grade 2 oligodendroglioma).

Many participants detailed how their beliefs about the extent to which they had accepted their diagnosis influenced their engagement in self-management. Acceptance was hindered by feeling a lack of direction, purpose, or control over one’s life, with ‘*slow progress*’ deterring their motivation to engage in self-management.

Most participants recounted the adverse effect of psychological distress on their ability to maintain a positive outlook. Some participants drew from positive aspects of their life (e.g. family) to maintain a positive attitude. However, the incurable nature of the condition, and the possibility of tumour progression, was ‘*mentally draining*’ and elicited anxiety and low mood in several participants. For many, this future uncertainty limited their perceived control, decision making, and engagement in goal and action planning, with some no longer comfortable thinking more than a few months ahead.

“When I am feeling down, I worry more about the impact [the tumour] has had, and the medication, the possible progression, and the impact it will have on my life in the future. And it is, kind of, mentally draining.” – Pa3 (aged 41–50, male, grade 2 oligodendroglioma).

Though most participants acknowledged the need for support, their self-confidence influenced whether they were motivated to seek support in a timely manner. Some perceived a stigma in asking for emotional support, saying that doing so ‘*feels like defeat*’. Others noted that the consequences of the tumour (e.g. cognitive deficits) impacted their ability to determine what support was needed.

“It’s hard for me to tell [what issues I’m having] because the very thing I’ve been measuring any side-effects with is the very thing that’s damaged.” – Pa35 (aged 41–50, male, grade 2 astrocytoma).

A few participants described how certain life events, and the success (or lack thereof) of previous attempts to self-manage, both influenced their continued engagement in self-management. For example, one participant recounted how a major life event (divorce) influenced by their diagnosis, was a considerable set-back to engaging with self-management. The importance of self-care was acknowledged by a few participants, though they also described low self-efficacy and unsuccessful attempts to engage in activities (e.g. exercise) at the desired level.

### Health status

The four factors that participants spoke about within ‘Health status’ were ‘*Co-morbidity*’, ‘*Illness severity*’, ‘*Symptoms/side-effects*’, and ‘*Cognitive functioning*’.

One participant was concerned that medication for a separate cancer diagnosis would be detrimental for their brain tumour. Further, though not explicitly a co-morbidity, some were unsure whether cognitive or mobility issues were a consequence of their condition or ageing, more generally. This led to uncertainty with whether, and how, to seek relevant information or support to facilitate self-management.

Some participants described conflicting feelings of luck when interpreting the consequences of their condition, comparing their experience to other people with brain tumours that were more impacted. Some reported feeling ‘*pretty much normal*’, citing an inability to drive as the main consequence of their condition. Others noted that symptom severity is unpredictable and variable, day-to-day, creating challenges for their active participation in self-management.

“Each day is completely different. Like today, I had a good night’s sleep it’s like, yeah, crack on with this. Yesterday I was like, “Ugh…” It’s just variable, pretty variable” – Pa19 (aged 51–60, male, grade 3 oligodendroglioma).

Many detailed the implications of the presence of, or anxiety about having, seizures, on social and occupational roles. Participants also outlined the influence of fatigue on self-management, describing how they required frequent breaks and rest to complete what they perceived as a simple task (e.g. sorting boxes). Some alluded to feeling inhibited by physical impairments, particularly those affecting mobility. Across each symptom, participants generally felt unable to do what they once could, hindering attempts to return to ‘normal’ living.

“I started to worry about getting up that early [for work] and whether the seizures would come back and all the rest of it. I found myself living a sort of lifestyle that I no longer wanted to be in because of the seizures.” – Pa35 (aged 41–50, male, grade 2 astrocytoma).

Most participants detailed an awareness that cognitive deficits had implications for their ability to self-manage, particularly concerning medication management (e.g. due to attention deficits) and activities of daily living (e.g. cooking due to memory deficits). Some reported the influence of communication deficits on their confidence to engage in social activities, reducing their opportunities for social engagement and connection.

“I don't think I would put my point across and join in the conversation as much as I used to because of [slurred speech].” – Pa38 (aged 51–60, female, grade 2 astrocytoma).

### Resources

The three factors that participants spoke about within ‘Resources’ were ‘*Financial*’, ‘*Equipment*’, and ‘*Psychosocial*’.

Almost all participants acknowledged the financial implications of their condition. For some, challenges with maintaining employment and accessing benefits resulted in considerable financial uncertainty.

“The housing or council benefit I can’t use that as one of my incomes. I can only work 16 h a week whereas I’d love to work more. If I did that, I’d have to lose the other benefits. If I lose those benefits and then [the tumour] does something I’d have to start right back at the beginning.” – Pa26 (aged < 40, female, grade 2 oligodendroglioma).

Others noted that for ‘*a chance to survive*’ substantial financial adjustments were needed to create an environment that enabled them to self-manage with a change in financial resources. A few participants described attempts to finance additional support (e.g. naturopath) themselves, but these were often unsustainable.

Some participants detailed how certain equipment and resources were helpful, for example: dosette boxes improved medication management; shoulder braces improved mobility impairments; and railcards and bus passes alleviated financial pressures concerning public transport.

“If you’re on anti-epileptic medication you can get the 20% disabled rail card for £20 a year or whatever it is. I’ve got a bus pass as well.” – Pa5 (aged 51–60, male, grade 2 oligodendroglioma).

Most participants emphasised the value of knowing that support was available from family and friends, should it be needed. Some participants highlighted that informal support networks were specifically important for maintaining social and occupational roles (e.g. transport to work). These participants spoke about how self-management would be more difficult if these support networks were not available.

“I have to travel to work, and it was just lucky I had friends around me that would give me a lift to work and giving me a lift back and stuff.” – Pa31 (aged 51–60, male, grade 2 oligodendroglioma).

However, sometimes participants experienced excessive or unsolicited support from others and this, when it occurred, limited their independence and opportunities to self-manage.

“People want to wrap you up in cotton wool and it’s like, “No. I just can’t walk as far as I used to.”.” – Pa18 (aged 51–60, female, grade 3 oligodendroglioma).

### Environmental characteristics

The three factors that participants spoke about within ‘Environmental characteristics’ were ‘*Home*’, ‘*Work*’, and ‘*Community*’.

A few participants mentioned the need for appropriate adjustments at home to accommodate for symptoms and impairments (e.g. walk-in shower for impaired mobility). One participant set up home gym equipment to provide a means of exercising.

“My bike is set up in the garage. I’ve worked a way of getting my leg over the top of it so I can pedal on it.” – Pa30 (aged > 60, male, grade 3 oligodendroglioma).

Several participants reported the effect of their work environment; for most, this concerned whether their employer was understanding and supportive, and made reasonable accommodations (e.g. allowing reduced working hours or additional breaks). Where understanding/support was lacking, work became a stressful, unpleasant environment for some; these participants felt that employers were scrutinising, rather than accommodating, changes in their capabilities. This created challenges for peoples’ ability to use support from employers in attempts to return to work.

“One of my managers wasn't particularly supportive of [a part-time arrangement] and started capability proceedings against me, which is very, very pleasant indeed – not.” – Pa15 (aged 51–60, Male, grade 2 astrocytoma).

Most participants detailed the influence of their community – essentially, where they lived – on their self-management. The (lack of) availability, and access to community support services (e.g. support groups) shaped access to information and skills to facilitate self-management. For some, this was exacerbated by an inability to drive to where support was available.

“I would have thought there would be [a support group] in my part of the country, the centres. This must be volunteers who are running it near to where it happened to them. If I could drive, I’d go every month, I definitely would.” – Pa19 (aged 51–60, male, grade 3 oligodendroglioma).

Several participants cited how good public transport links facilitated access to support and activities of daily living (e.g. grocery shopping); however, due to risk of seizures or cognitive difficulties with planning, public transport was not seen as a viable option for some.

### Healthcare system

The three factors that participants spoke about within ‘Healthcare system’ were ‘*Access*’, ‘*Navigating system/Continuity of care*’, and ‘*Relationship with provider*’.

Many participants described how access to support and information from healthcare professionals and the healthcare system influenced their ability to self-manage. Some spoke about how being able to easily access their clinical care team provided opportunities to acquire knowledge and support.

“The nurses, you can ring them anytime. I’ve got their times and their numbers pinned up in the kitchen there, so, that is like a little support team in itself so that’s useful.” – Pa28 (aged > 60, male, grade 2 astrocytoma).

However, several participants reported the need to seek information elsewhere because ‘*there’s nothing (provided) upfront*’, or they had received too much information at a time when it was not useful. For some, a lack of knowledge about, or access to, available support within the public healthcare system meant they sought private alternatives.

The majority of participants reported unsuccessful attempts to navigate the healthcare system; markedly several participants noted the absence of advice and signposting to available support. Some suggested they needed and wanted to be ‘*given the tools*’ to self-manage but support received was insufficient; sometimes care ceased when it was still needed.

“They put me on some physio, but I only had, maybe, six sessions and then the physiotherapist left, and it wasn’t really continued…if there had been a handover to someone else, I think that would have been much more productive for myself. I had maybe two years where I wasn’t really doing anything.” – Pa26 (aged < 40, female, grade 2 oligodendroglioma).

When navigating non-healthcare services (e.g. social welfare system, vehicle licencing authorities), many participants expressed challenges with understanding the health-related information required (e.g. what treatment(s) for the tumour they had received), creating setbacks when attempting to arrange additional support and self-manage.

Most participants recounted the influence of their relationship with healthcare providers, reporting that the strength of the relationship depended on the provider’s social skills. Several participants detailed feeling trust in, and reassurance from, healthcare providers, which provided them with the knowledge and belief that they could self-manage.

“I see a psychiatrist who’s in the cancer centre. And he’s absolutely fantastic. And he’s always really, really good at being able to give me advice about what I can do if I’ve got a problem.” – Pa25 (aged 41–50, male, grade 2 oligodendroglioma).

Conversely, where participants reported negative interactions, this often exacerbated health-related concerns, as they were unable to acquire the appropriate knowledge to facilitate self-management.

## Discussion

Self-management can have numerous quality-of-life, clinical, and health economic benefits [3, 4], but various factors can influence engagement in self-management [5]. Our study aimed to identify and understand the barriers and facilitators to self-management in people with LGG, a group who may experience a wide-range of chronic, tumour-related, symptoms and impairments.

In accordance with Schulman-Green et al.’s framework of factors influencing self-management [5], our data extensively supported all five categories, encompassing all 18 factors, and highlights how these factors distinctively influence self-management in people with LGG. What was also evident from our findings was that numerous factors may interact to influence an individual’s ability to self-manage. For example, with support group attendance, the individual may need self-confidence to seek support (‘*Motivation*’ within ‘Personal/lifestyle characteristics’) to then receive appropriate signposting to available support (‘*Navigating system*’ within ‘Healthcare system’). Should a support group be available in their location (‘*Community*’ within ‘Environmental characteristics’), transport issues or risk of seizures may mean they feel unable to attend alone (‘*Psychosocial*’ within ‘Resources’). Moreover, communication impairments might inhibit self-confidence to contribute to the support group (‘*Cognitive functioning*’ within ‘Health status’), which may precede the initial motivation to seek support. Hence, challenges with self-management can be complex and multi-dimensional, and this must be considered in the development of self-management support.

Our findings also highlight the adverse influence of beliefs (e.g. lack of control) and psychological distress on self-management. Though we draw similarities to studies in other cancers [16, 33], we would argue that people with LGG may be somewhat distinct in living with an incurable, life-limiting illness; indeed, future uncertainty concerning possible tumour progression was described as the main source of psychological distress in our participants, congruent with Ley et al. [24]. Further, challenges with acceptance and future uncertainty led to difficulties with maintaining a positive outlook and feeling motivated to self-manage. This could have implications for whether an individual seeks support for, and engages in, self-management; hence, this is an important consideration for healthcare professionals, when implementing self-management support for people with LGG.

Quantitative studies of people with LGG have indicated that poor cognitive function and seizure burden are consistently associated with worse health-related quality-of-life [23]. We expand on this to highlight how such symptoms and impairments can create specific challenges for self-management engagement (e.g. the often-significant cognitive deficits can hinder medication management). These support needs are somewhat distinct from the influence of symptoms on self-management found in studies of other cancers [14, 17] and have more in common with neurological populations such as MS or stroke [11, 34]. Therefore, when encouraging people with LGG to self-manage, consideration of their cognitive function and seizure burden will help determine whether certain self-management activities (e.g. cooking, physical activity, use of public transport) are achievable, or require an adapted approach. Implementation of needs assessments in clinical practice could be of value to identify issues or problems that people with LGG would like more support with; in this way, support could be tailored to the needs of the individual. For example, identifying that an individual would like help with managing cognitive deficits could prompt the co-development of self-management strategies (e.g. use of external aids) to overcome these challenges. Existing reviews show that many such needs assessment instruments are available for people with cancer [35, 36], though none of these seem to be specific to people with LGG or brain tumours more generally.

Since LGGs are typically diagnosed in working-aged adults [19], those affected may want or need to return to work [37]. Our data highlights the importance of understanding and support from employers for work-related self-management. Still, several factors may interact (including ‘*Symptoms/side-effects*’ and ‘*Psychosocial resources*’) to influence people with LGGs’ work experiences [38]. For example, transport assistance facilitated engagement in occupational roles; one of numerous ways that psychosocial resources aided self-management. This is consistent with the wide-ranging role and responsibilities of family and friends (e.g. cognitive, emotional, practical support) that we have observed in a parallel set of interviews with informal caregivers of people with LGG. Informal caregivers also have a critical role in supporting and facilitating self-management [39]; however, care needs to be taken – as we highlight here, and as reported by others – to ensure such support does not tip over into limiting the independence of people with LGG [40], as this could create a barrier to self-management.

Participants in our study reported difficulty with accessing appropriate support; as with Langbecker et al.’s study of people with brain tumours [25], this barrier was exacerbated by whether support was available and accessible within a person’s community. Studies of people with chronic illness detail the impact of transport challenges on self-management [10, 41]. However, people with LGGs’ experiences are distinct, at least in cancer terms, because their driving licence is typically revoked, often due to ongoing seizure activity, presenting consequent challenges (e.g. time and uncertainty) with reobtaining their licence. Hence, this barrier to self-management may be sustained longer-term for people with LGG, meaning greater support with transport challenges may be necessary, particularly for those with weaker support networks.

Our participants described the need to actively engage in help-seeking to ensure awareness of available support, as insufficient or inappropriate information, advice, and signposting from healthcare providers, was a key barrier to self-management. However, help-seeking may be hindered by poor knowledge [7], as not knowing what to expect (e.g. symptoms) meant our participants often did not seek appropriate, timely support. Further, our data supports the suggestion that people with brain tumours can underestimate cognitive, emotional, psychological, and social changes [42]. Nonetheless, with sufficient knowledge, self-confidence to seek help is also important for self-management [9], and – as our data suggests – this may be influenced by the person with LGG’s relationship with their healthcare provider. We indicate the benefits (e.g. reassurance) of strong relationships, and detriments (e.g. distrust) of poor relationships between people with LGG and their healthcare provider. Consideration of how others might facilitate or encourage autonomy within supportive relationships could have fundamental importance for improving peoples’ confidence to self-manage.

### Implications

Internationally, there is a call to action for health systems to improve integration of self-management support in cancer care [43]. In a recent systematic review of self-management interventions in cancer, it was noteworthy that none were targeted to people affected by brain tumours [3]. The present analysis comprehensively complements and expands on data we have reported elsewhere from this study (e.g. [26].), filling the evidence gap around self-management and its determinants among people with LGG; this is a fundamental first step towards developing and/or implementing effective self-management support for this population [44]. Overall, our findings serve to improve awareness of the challenges faced by people with LGG that may influence whether they are able to self-manage in day-to-day life, while emphasising how these challenges can co-occur and vary for each individual. For healthcare professionals, who are increasingly encouraged to engage patients with self-management, and researchers interested in developing self-management interventions for those affected by cancer, such an understanding is invaluable.

### Strengths and limitations

A key strength of our study is the novel understanding of factors influencing self-management in people with LGG; semi-structured interviews provided the freedom to explore these factors across a diverse range of contexts (e.g. domestic and social roles). We are confident that reasonable data sufficiency was achieved, as there was extensive data, supported by multiple quotes, to understand the factors influencing self-management in people with LGG.

Due to Covid-19, all interviews were conducted remotely; this facilitated recruitment across the UK [45], and may have encouraged more disclosure, through less discomfort and a perception of greater anonymity [46]. However, despite attempts to support participation of people with LGG with cognitive and communication impairments, remote interviews and expected interview length (approx. 90 min) may have made it more difficult for them to take part. It is not uncommon in LGG literature for people with these impairments to be excluded [23], therefore, further consideration of how to support participation is required (e.g. multiple, shorter interviews to mitigate the risk of fatigue).

We sought a wide range of times since diagnosis in our sample to generate an understanding of the factors influencing self-management in people living short- and long-term with an LGG. The challenges perceived by someone more than 10 years post-diagnosis are likely different to the challenges perceived in the early stages following primary treatment. However, the cross-sectional design means we cannot be certain whether or how these factors may be experienced differently over time. Future longitudinal studies could be beneficial to explore how barriers and facilitators to self-management in people with LGG may change over time.

## Conclusions

This study explored the barriers and facilitators to self-management in people with LGG, highlighting the distinctive experiences within the wide-ranging factors influencing self-management in this population. These findings may improve awareness of the challenges faced by people with LGG in self-management following completion of initial treatment. Notably, we emphasise potential supportive care needs, and how multiple factors may interact, and influence each individual differently. Our findings could be useful to inform the development of self-management interventions for people with LGG, ensuring, where possible, that potential barriers are addressed to facilitate effective engagement in self-management.

## Supplementary Information

Below is the link to the electronic supplementary material.

Supplementary file1 (PDF 146 KB)

## Data Availability

The data that support the findings of this study may be available from the Chief Investigator (Professor Linda Sharp; linda.sharp@ncl.ac.uk) upon reasonable request.
